# The Redox Balance and Membrane Shedding in RBC Production, Maturation, and Senescence

**DOI:** 10.3389/fphys.2021.604738

**Published:** 2021-02-16

**Authors:** Eitan Fibach

**Affiliations:** Department of Hematology, Hadassah University Hospital, Jerusalem, Israel

**Keywords:** red blood cell, microvesicles, membrane, aging, oxidation stress

## Abstract

Membrane shedding in the form of extracellular vesicles plays a key role in normal physiology and pathology. Partial disturbance of the membrane–cytoskeleton linkage and increased in the intracellular Ca content are considered to be mechanisms underlying the process, but it is questionable whether they constitute the primary initiating steps. Homeostasis of the redox system, which depends on the equilibrium between oxidants and antioxidants, is crucial for many cellular processes. Excess oxidative power results in oxidative stress, which affects many cellular components, including the membrane. Accumulating evidence suggests that oxidative stress indirectly affects membrane shedding most probably by affecting the membrane–cytoskeleton and the Ca content. In red blood cells (RBCs), changes in both the redox system and membrane shedding occur throughout their life—from birth—their production in the bone marrow, to death—aging in the peripheral blood and removal by macrophages in sites of the reticuloendothelial system. Both oxidative stress and membrane shedding are disturbed in diseases affecting the RBC, such as the hereditary and acquired hemolytic anemias (i.e., thalassemia, sickle cell anemia, and autoimmune hemolytic anemia). Herein, I review some data-based and hypothetical possibilities that await experimental confirmation regarding some aspects of the interaction between the redox system and membrane shedding and its role in the normal physiology and pathology of RBCs.

## Introduction

Most, if not all, cells shed part of their plasma membrane as extracellular vesicles, the role of which in physiological and pathophysiological processes is increasingly appreciated (Raposo and Stoorvogel, 2013). These vesicles differ in their mechanisms of production, composition, and function. In red blood cells (RBCs), membrane shedding (vesiculation) continues throughout their entire life, from birth to death, serving multiple roles at different phases: production and maturation in the bone marrow, circulation, functioning, and aging (senescence) in the peripheral blood, and removal (clearance) in sites of the reticuloendothelial system, such as the spleen (Leal et al., 2018). Oxidative stress is one of the mechanisms that have been suggested to contribute to vesiculation (Willms et al., 2016, 2018).

The redox status of cells depends on the equilibrium between oxidants and antioxidants. Its homeostasis is crucial for the functioning of many systems of the body, including the normal physiology of RBCs and their ability to produce and shed membrane-bound vesicles (Margolis and Sadovsky, 2019). Oxidative stress, which presents an imbalance in the redox state, is considered to be involved in many diseases, including those intimately concerned with the erythroid cell system, such as the hemolytic anemias (Fibach and Rachmilewitz, 2008).

Herein, I review the interdependence of oxidative stress and membrane shedding throughout the life of the RBCs and their involvement in normal and pathological physiology.

## Membrane Shedding

Membrane shedding in the form of membrane-bound particles is common to all cells. It occurs as exosomes (50–150 nm in diameter)—released upon exocytosis; microvesicles (MVs) or microparticles, and apoptotic bodies (1–5 μm)—generated by blebbing of the plasma membranes of cells undergoing programmed cell death (Raposo and Stoorvogel, 2013).

The RBC, like any other cell, produces MVs, which are shed from the plasma membrane (Willekens et al., 2008; Leal et al., 2018). This process occurs at all stages of the RBC life: its production, maturation, aging (senescence), and removal, and it constitutes an intrinsic part of homeostasis in terms of the quantity and quality of the RBC. It also occurs during the storage of RBC in banks. Normal blood plasma contains approximately 1,000 RBC-derived MVs per milliliter; however, as they contain immunological recognition and removal signals, they are rapidly eliminated (probably within minutes) from the circulation by the spleen macrophages (Willekens et al., 2008).

Microvesicles are surrounded by a lipid-bilayered membrane that is different in its composition from the plasma membrane from which they are derived, suggesting that their formation (vesiculation) involves a regulated sorting process, leading to enrichment or depletion of various components. MVs are enriched in cellular constituents characterizing the oldest RBC—the glycated hemoglobins (Hb), HbA1c and HbA1e2 (Leal et al., 2018), and redox enzymes, such as glutathione S-transferase, thioredoxin and peroxiredoxins 1 and 2, and the protein-degrading ubiquitin. They contain senescence signals such as phosphatidylserine (PS), and band-3 epitopes (Westerman and Porter, 2016). In addition, MVs contain the glycosylphosphatidyl-inositol (GPI)–anchored, complement-inhibiting proteins, the decay-accelerating factor (CD55), and the membrane inhibitor of reactive lysis (CD59), although probably not in a functional form, as demonstrated for the GPI-anchored enzyme acetylcholinesterase (Leal et al., 2017).

Membrane shedding affects the production of RBCs, their functionality and their clearance. In the circulation, the process leads to the removal of damaged cell constituents. This is exemplified by the loss of 20% of the Hb and the cell membrane during RBC aging (Leal et al., 2018).

## Mechanisms of Membrane Shedding

Two mechanisms have been suggested as the main driving forces of membrane shedding and extracellular MV production:

### Partial Cytoskeleton:Membrane Uncoupling

The RBC membrane comprised a cytoskeleton and a lipid bilayer. The latter includes various phospholipids and sphingolipids, cholesterol, and integral membrane proteins such as Band-3 and glycophorin. The cytoskeleton is tethered to the lipid bilayer via “immobile” Band-3 proteins at the spectrin–ankyrin binding sites and via glycophorin at the actin junctional complexes. One mechanism of vesiculation occurs as a result of phosphorylation of the membrane proteins, mostly Band-3, leading to a weakening of the link between the lipid bilayer and the cytoskeleton [for detailed review, see Leal et al. (2018)]. This hypothesis is supported by the finding of accumulated amounts of Band-3 and actin, but the absence of spectrin, in MVs.

### Calcium Accumulation

Calcium is a potent, specific, and tightly controlled cellular regulator including in RBC [for detailed review, see Bogdanova et al. (2013)]. Increased intracellular Ca^2+^ concentration could be triggered by various cytotoxic stimuli such as hyperosmolarity, exposure to xenobiotics, and oxidative stress (Kass et al., 1990; Cameron et al., 1993) that occurs during normal RBC senescence and eryptosis (apoptosis-like pathological destruction of mature enucleated RBCs) (Lang et al., 2005). The increase in Ca^2+^ triggers biochemical and structural changes that result in vesiculation, such as activation of the apoptosis mediator caspase-3, which can trigger vesiculation, and enhancing Floppase and scramblase and inhibiting Flippase—the enzymes that control the externalization of PS (Allan and Thomas, 1981). Vesicles collected under experimental treatment with Ca^2+^ ionophores, such as A23187, are of two sizes (∼200 and ∼120 nm in diameter) that contain Hb and are enriched in GPI-anchored proteins (e.g., acetylcholinesterase and CD55) and raft lipids, but are free of cytoskeleton components (Leal et al., 2018).

These two mechanisms may not be mutually exclusive. For example, increased Ca^2+^ level may activate proteolytic enzymes such as calpains (Guerini et al., 2005) that decrease the cytoskeleton:membrane interaction, leading to vesiculation.

Although these mechanisms are most probably involved in vesiculation, they may not be the primary events of the process. For example, cytoskeleton modifications may be the consequence, rather than the cause, of the vesiculation (Guerini et al., 2005). It was suggested that, in the blood bank, only after 35 days of storage RBCs exhibit clustered Band-3 MVs (Azouzi et al., 2018), which could imply that another mechanism is involved in vesiculation before that. Based on the difference in the protein composition of MVs generated upon senescence and storage to those induced experimentally by Ca^2+^ ionophores, Bosman et al. proposed that the latter is not the initiating event of the generation of RBC-MVs (Bosman, 2013).

## Oxidative Stress

The redox status represents the balance between oxidants, such as reactive oxygen species (ROS), and antioxidants [for review, see Halliwell and Gutteridge (1999)]. Imbalance due to excess oxidants or insufficient antioxidants results in oxidative stress. Although oxidants have important roles in normal physiology, e.g., in signal transduction, excess oxidants may interact with, and damage, various cellular components (e.g., proteins, lipids, and nucleic acids). Aging of the organism; senescence of cells, such as the RBCs; and many environmental and pathological situation are related to oxidative stress. Oxidative stress is involved in many pathologies such as neurodegenerative, cardiovascular, metabolic, and malignant diseases. In some diseases, the RBCs are the prime target of oxidative stress; this includes the hemolytic anemias (Fibach and Rachmilewitz, 2008).

In most cells, ROS is generated during oxidative energy production in the mitochondria. Approximately 2% of the total O_2_ consumption results in the superoxide anion radical (O${}_{2}^{•}$) (Halliwell and Gutteridge, 1999). In the mitochondria-deficient RBC, Hb is the primary source of ROS generation, when heme iron interacts with oxygen. Normally, approximately 3% of the Hb is auto-oxidized to form metHb and superoxide that in turn produces hydrogen peroxide (H_2_O_2_) and oxygen by dismutation (Scott et al., 1989).

Many pathological conditions are associated with oxidative stress in various cell types (Liguori et al., 2018). We have studied particularly the hereditary and acquired hemolytic anemias, where oxidative stress involves mainly, but not exclusively, the erythroid cells (Fibach and Rachmilewitz, 2008). In these diseases, the destruction of mature RBCs and their precursors (hemolysis) is accelerated—over the capacity to produce new ones, resulting subsequently in chronic anemia. Among the hemolytic anemias are the following diseases:

### The Hemoglobinopathies—Thalassemia (Thal) and Sickle Cell Disease (SCD)

Are caused by hereditary mutations in the globin genes. The major adult Hb, HbA, is a tetramer of two α-globin and two β-globin chains (α_2_β_2_). In α-thal or β-thal, the respective genes are mutated, leading to reduced or no production of their polypeptides (Rund and Rachmilewitz, 2005). In SCD, a mutation in the β-globin gene leads to the production of abnormal globin chains (β^S^) that form the abnormal sickle Hb (HbS). The latter polymerizes under the deoxygenated conditions of the narrow capillaries, altering the physical and chemical properties of the RBCs. They acquire a sickle-like morphology and an increased tendency to hemolyze and to adhere to the vasculature walls (Steinberg, 1998; Cisneros and Thein, 2020).

### Glucose-6-phosphate Dehydrogenase Deficiency

An RBC enzymopathy caused by mutations in the Glucose-6-Phosphate Dehydrogenase (G6PD) gene. The latter encodes a key enzyme of the pentose pathway (hexose monophosphate shunt), which in RBCs is the major supplier of the reducing agent nicotinamide adenine dinucleotide phosphate (NADPH) that is essential for several biosynthetic pathways (Luzzatto and Battistuzzi, 1985) and the redox homeostasis (Tian et al., 1998). The G6PD- and NADPH-deficient RBCs are under oxidative stress, especially after oxidative insult induced by certain foods (fava beans) and oxidative drugs, oxidant drugs, or chemicals (Arese et al., 2012).

### Hereditary Spherocytosis

An RBC membranopathy due to mutations in various genes encoding for membrane proteins. Deficiency in spectrin is the most prevalent abnormality (Panizo Morgado et al., 2020), but sometimes it is secondary to a deficiency or dysfunction of the spectrin-binding protein–ankyrin (Hanspal et al., 1991; Panizo Morgado et al., 2020). The disease is characterized by RBCs with sphere shape (instead of biconcave disks), with reduced surface-to-volume ratio and increased osmotic fragility—properties that make them inclined to hemolysis (Perrotta et al., 2008; Chagula et al., 2020). Elliptocytosis is another RBC membranopathy with spectrin tetramer self-association, leading to RBCs with an elliptical or elongated shape (Pollet et al., 2020).

### Paroxysmal Nocturnal Hemoglobinuria

A clonal stem cell disorder due to acquired somatic mutations predominantly in the phosphatidylinositol glycan complementation class A gene. The gene encodes an enzyme that participates in the production of the GPI anchor, by which some proteins are linked to the plasma membrane. Lack of this anchor leads to a partial or complete deficiency in these proteins on the hematopoietic stem cells and their differentiated progeny. The decay-accelerating factor (CD55) and the membrane inhibitor of reactive lysis (CD59) (Walport, 2001) belong to this family, making the affected RBCs prone to hemolysis by complement fixation and activation (Parker, 2007).

### Autoimmune Hemolytic Anemia

An acquired disease caused by autoantibodies to surface RBC antigens. Binding of these antibodies marks them for complement-mediated and/or Fc-mediated lysis—intravascular and extravascular hemolysis, respectively. This can occur alone or with other conditions such as autoimmunity, malignancy, pharmacological treatment, blood transfusion, and pregnancy (Sawitsky and Ozaeta, 1970).

### Myelodysplastic Syndromes

These conditions, affecting mostly elderly people, are characterized by ineffective production (dysplasia) of several blood cell lineages and bone marrow failure, leading to severe anemia that may require blood transfusion. One-third of the patients develop fatal acute myelogenous leukemia (Cazzola, 2012).

These, and other hemolytic anemias, vary in their etiology, but they all share damage to erythroid cells by oxidative stress [for review, see Fibach and Rachmilewitz (2008)]. Using flow cytometry methodology, we have documented oxidative stress in their RBCs that included high production of ROS and membrane lipid peroxides and low content of reduced glutathione (Freikman et al., 2008), their primary antioxidant. ^1^H–nuclear magnetic resonance (NMR) analysis demonstrated their oxidative stress by an elevated lactate-to-pyruvate ratio (Freikman et al., 2008).

Oxidative stress in RBCs in these anemias is due mainly, but not exclusively, to the following reasons:

### Hemoglobin Instability

In thal, because of the shortage of one globin chain, there is an excess of other globins α in β-thal and γ and β in α-thal. These unmatched globins form homotetramers that dissociate into monomers that are oxidized; first to meHb and then to hemichromes (Rachmilewitz, 1974). Heme, iron, and the degraded protein moiety accumulate in the cytosol and on the plasma membrane. This leads to increased generation of ROS, catalyzed by free iron (see below), which damage membrane lipids and proteins, e.g., Band 4.1 and Band-3 (Rachmilewitz and Harari, 1972; Advani et al., 1992). In SCD, the metHbS is more unstable than metHbA, resulting in increased production of hemichromes that precipitate as Heinz bodies (Winterbourn and Carrell, 1974).

### Iron Excess (Iron Overload)

In most hemolytic anemias, iron accumulates as a result of excess acquisition due to RBC transfusions and increased absorption from the diet (Fibach and Rachmilewitz, 2008). In addition, the release of iron-containing compounds (Hb or hemin) during hemolysis adds to the iron load. Normally, iron circulates complexed to transferrin and is taken up by cells through the membrane transferrin receptor (TfR) (Wang and Pantopoulos, 2011). Most of the intracellular iron is bound in a redox-inactive form to proteins such as in Hb and myoglobin; excess iron is stored in ferritin (Konijn et al., 1979). When extra iron is acquired, it saturates the transferrin; excess iron exists in the plasma as non–transferrin-bound iron (Breuer et al., 2000). This iron is taken up by cells through transferrin-independent pathways and forms the “labile iron pool” (Fibach and Rachmilewitz, 2010; Prus and Fibach, 2011)—a transitory intermediate between the cellular iron pools (Jacobs, 1977). Labile iron is redox-active—it takes part in the Fenton and Haber–Weiss reactions that generate ROS (Fibach and Rachmilewitz, 2010).

### Complement Activation

Serum complement and oxidative stress are associated with paroxysmal nocturnal hemoglobinuria (Amer et al., 2008) and autoimmune hemolytic anemia (Fibach and Rachmilewitz, 2008). The affected RBCs are hypersensitive to oxidative insults (e.g., by hydrogen peroxide), and their oxidative status increases by interaction with activated complement before hemolysis. Antioxidants, such as N-acetyl cysteine and ascorbic acid (vitamin C), were found to reduce excess hemolysis *in vitro* (Amer et al., 2008), and the antioxidant food supplement, fermented papaya preparation, *in vivo* (Ghoti et al., 2010).

## Oxidative Stress and Membrane Shedding

Several findings favor the primary involvement of oxidative stress in vesiculation (Szigyarto et al., 2018; Nader et al., 2020): (I) MVs are enriched in antioxidant enzymes and irreversibly oxidized Hb (Sudnitsyna et al., 2020); (II) some studies have indicated that treatment with oxidants decreased vesiculation, whereas antioxidants have an opposite effect (Stowell et al., 2013; Nader et al., 2020). Oxidative stress, through the effects of ROS, may trigger both mechanisms of vesiculation—the clustering of Band-3 and the accumulation of intracellular Ca^2+^. The effect on Band-3 involves (I) activation of Src tyrosine kinases that phosphorylates Band-3; (II) oxidation of Hb into hemichromes that interact with the Band-3 cytoplasmic tail. In both cases, these results in clustering and mobility of Band-3 by detachment from the membrane skeleton, likely by release from ankyrin (Azouzi et al., 2018). As for Ca^2+^, virtually every cellular Ca control mechanism is both affected by oxidative stress and is able to affect it (Leal et al., 2017). The cytosolic content of Ca^2+^ is increased by oxidative stress through the effects on Ca pumps, exchangers, channels, and binding proteins (Leal et al., 2017).

## Externalization and Shedding of Phosphatidylserine

Membrane shedding and its modulation by oxidative stress are intimately related to the externalization and shedding of PS. The distribution of phospholipids across the plasma membrane of all cells, including the RBCs, is asymmetrical (Zwaal et al., 2005); aminophospholipids, e.g., PS, are preferentially present in the inner leaflet, whereas lipids with a choline head, e.g., phosphatidylcholine (PC), are mainly present in the outer leaflet of the membrane (Op den Kamp, 1979). The PS distribution across the membrane is under a dynamic equilibrium. Inward movement is catalyzed by the enzyme aminophospholipid translocase, whereas the outward movement, by the scramblase. Some of the external PS is shed to the extracellular environment. One mechanism of oxidative stress–mediated effect on PS externalization and shedding is by inhibition of the aminophospholipid translocase, causing the PS to “flip-flop” from the inner to the outer leaflet of the membrane (Pattanapanyasat et al., 2004).

We studied the interrelationship between oxidative stress on PS externalization and shedding in RBCs and their precursors by two methodologies: flow cytometry and NMR spectroscopy. Parameters studied by flow cytometry included the generation of ROS and membrane lipid peroxides and the contents of reduced glutathione (Amer and Fibach, 2004; Amer et al., 2004), labile iron pool (Prus and Fibach, 2008), and Ca^2+^ (Freikman et al., 2011). To measure the cellular distribution and shedding of PS, we designed a two-step fluorescence inhibition procedure (Freikman et al., 2009). Commonly, PS is measured by a fluorochrome-conjugated, PS-specific, protein–annexin V. In most studies, the method refers to the percentage of cells expressing a high level of PS (Alaarg et al., 2013; Ferru et al., 2014), neglecting cells with less bound annexin V, giving the impression that PS externalization is an “all or none” process. This measurement is mainly applicable to populations with a significant percentage of highly positive cells (e.g., following induction of apoptosis). However, *in vivo*, because of their short survival, very few such cells exist, making their determination unreliable. Additionally, this procedure does not measure the inner PS (unless the cell plasma membrane is permeabilized), nor the shed PS. Most importantly, the procedure yields a relative (in mean fluorescence channel) rather than absolute quantitative values. To overcome these shortcomings, we devised a protocol that entails two steps. First, the PS on the surface of intact cells, or in cell lysates, supernatants, or blood plasma is bound to an excess amount of annexin V. Then, the residual, non-bound annexin V is quantified by binding to PS exposed on apoptotic cells (e.g., 6-day old HL-60 cells), serving as an indicator reagent, the fluorescence of which is inversely related to the PS in the tested sample (Zwaal et al., 2005).

Using this methodology, we confirmed that mature RBCs and their precursors in thal, as in other hemolytic anemia (see below), are under oxidative stress. The results also demonstrated that oxidatively stressed RBCs (old vs. young, thal vs. normal) have a lower content of total cellular PS but more exposed and shed PS. This was reflected by a moderate increase in the proportion of highly annexin V–positive cells and by a significant increase in the average (mean fluorescence channel) cellular PS exposure of the entire population. The increased PS shedding by thal RBCs was also reflected in the higher PS concentration found in sera from patients versus normal donors. Interestingly, in addition to PS in MVs (Pattanapanyasat et al., 2004), we found significant amounts of PS shed in a membrane-free form (Freikman et al., 2008, 2009).

The interrelationship of the oxidative status, Ca flux, and PS shedding was demonstrated by measuring PS and Ca^+2^ in stressed and non-stressed RBCs (Freikman et al., 2011). Treating of thal RBCs with antioxidants (e.g., ascorbic acid or N-acetyl cysteine) led to decreased external and shed PS (Freikman et al., 2009), while treating normal RBCs with oxidants, the Ca ionophore A23187, or increasing the Ca concentration of the medium concomitantly increased the oxidative state, the Ca flux, and the PS shedding (Freikman et al., 2009).

These results were confirmed by NMR spectroscopy (Freikman et al., 2008). ^1^H-NMR showed a higher ratio of lactate/pyruvate, reflecting oxidative stress, in normal RBCs treated with oxidants. ^31^P-NMR showed more PC and less PS in the thal RBCs that were reversed by antioxidants. When RBCs were incubated in phosphate-buffered saline, more PS was found in the supernatant of thal cells than of normal cells. Antioxidants reduced shedding of PS, whereas oxidants increased it. The plasma of thal patients contained more PS and less PC than normal plasma. These results confirmed the results obtained by flow cytometry, indicating that the decreased PS on the membrane of oxidatively stressed RBCs resulted from increased shedding. The shed PS was further analyzed in MVs from purified from the plasma and from RBC supernatants. The results indicated more PS in thal MVs than in their normal counterparts.

These changes in the membrane composition increase the osmotic resistance and the susceptibility of RBCs to undergo phagocytosis. Using an *in vitro* system, we have shown that inducing PS externalization by treatment with an oxidant increased erythrophagocytosis by macrophages, whereas adding PS prevented it. The latter effect was most probably due to a competitive binding to PS receptors on the macrophages (Freikman et al., 2009).

## Membrane Shedding in RBC Diseases Associated With Oxidative Stress

In addition to oxidative stress, hemolytic anemias are characterized by increased MVs derived from RBCs and their precursors as well as in other blood cell types and platelets (Westerman and Porter, 2016). Oxidative stress might be the driving force of their increased production, in addition to other disease-specific mechanisms [for review, see Leal et al. (2018)].

In α- and β-thal, the hemichromes produced in the mature RBCs and their Hb-containing precursors due to the production of unbalanced globin chains and homo-tetramers bind to Band-3 and induce their dimerization. Band-3 is subsequently phosphorylated by tyrosine kinases, weakening the cytoskeleton/membrane interaction, resulting in vesiculation.

In SCD, RBCs, platelets, and polymorphonuclear neutrophils exhibit oxidative stress (Amer et al., 2006), which in RBCs is due to the deoxygenated HbS polymers and other factors (e.g., the chronic inflammatory state) (Fibach and Rachmilewitz, 2008). It damages the RBC membrane proteins and lipids and contributes to increased intracellular Ca^2+^ and tyrosine phosphorylation of Band-3 (Evans et al., 1984). These changes lead to dehydration and membrane rigidity, poor deformability, and destabilization, leading to the increased vesiculation (Westerman and Porter, 2016). The MVs are related to the pathogenesis; they are high during both steady-state and painful crisis conditions (Margolis and Sadovsky, 2019).

In hereditary spherocytosis, although oxidative stress is common to RBCs (as well as other blood cell types) (Ghoti et al., 2011), the underlying defect, i.e., changes in ankyrin, spectrin, or Band-3, might affect the vesiculation process, producing MVs of different compositions. For instance, Band-3 has been found in MVs from ankyrin- or spectrin-defective RBCs, but not from those defective in Band-3 (Reliene et al., 2002).

Paroxysmal nocturnal hemoglobinuria and autoimmune hemolytic anemia, which are associated with oxidatively stressed RBCs (Amer et al., 2008; Fibach and Rachmilewitz, 2008), also demonstrated changes in vesiculation (Pan et al., 1991; Leal et al., 2019).

In summary, membrane shedding is elevated in oxidatively stressed RBC–centered diseases. Defects caused by oxidative stress in the cytoskeleton/membrane association are most likely the main underlying mechanisms, although other disease-related changes may contribute to vesiculation as well. RBC-derived MVs may have beneficial effects, such as preventing the untimely removal of functional RBCs. On the other hand, they may be actively involved in pathology, e.g., by their effects on inflammation, thrombosis, and autoimmune reactions (Fibach and Rachmilewitz, 2008). This double-edged effect emphasizes the need for a better understanding of their mechanisms of generation, their pathophysiological consequences, and modes of prevention.

## The Redox Balance and Membrane Shedding During RBC Life

Changes in the redox state and the membrane shedding characterize the RBCs at different stages of their life from birth to death, serving specific roles at each stage. The redox state depends on the rate of ROS production and scavenging (Halliwell and Gutteridge, 1999). In erythroid progenitors and precursors, ROS are produced at a high rate mainly as a byproduct of energy production in the mitochondria. They are reduced considerably in the mitochondria-free mature RBCs where they are produced mainly as a result of oxygenation of Hb. Upon senescence, RBCs undergo oxidative stress mainly due to a decrease in the antioxidative defense effects of the enzymes superoxide dismutase, catalase, G6PD, and aspartate aminotransferase (Kumar and Rizvi, 2014). The latter two enzymes are involved in the formation of antioxidant reduced glutathione and NADPH (Fornaini et al., 1985).

We have studied membrane shedding during RBC generation and senescence with respect to the externalization and shedding of PS (Freikman and Fibach, 2011).

## Membrane Shedding in the Bone Marrow

In humans, RBC production (erythropoiesis) takes place in the bone marrow by a physiologically regulated process that entails the proliferation and maturation of erythroid progenitors and precursors. Membrane shedding may participate in the following processes:

### Erythropoiesis

The extent of erythropoiesis depends on the relative rates of generation of erythroid-committed progenitors from the pluripotent hematopoietic stem cells, their proliferation, and maturation to morphologically identified precursors and eventually to reticulocytes. These processes are taking place mainly in the bone marrow and are regulated by the hematopoietic environment and various glycoprotein cytokines. The latter induce signal transduction pathways initiated by interaction with specific surface receptors. Among the cytokines, the erythroid hormone, erythropoietin (Epo), serves a particularly important role. It stimulates the proliferation and maturation (Palis, 2014) and suppresses the death of erythroid precursors, serving as an antiapoptotic agent (Testa, 2004). The extent of the effect of cytokines is proportional to their concentration in the bone marrow environment and the abundance (number/concentration) of the surface receptors on the affected cells. Epo is produced mainly in the kidneys in response to the oxygen tension of its tissue; hypoxic conditions, such as at high altitudes or anemia, stimulate Epo production, leading to its high levels in the bone marrow. Its surface receptors (EpoR) are modulated during erythroid maturation, in a biphasic mode. Their abundance peaks in the late erythroid progenitors (colony-forming units—erythroid) and then gradually decreases to disappear altogether from mature RBCs (Wickrema et al., 1991). This modulation may explain the increasing effect of Epo during progenitor maturation and its diminishing effect on the more mature precursors. Several groups suggest that some RBCs do carry low levels of EpoR, which may explain the effect of Epo on mature RBCs (Amer et al., 2010). It could be hypothesized that membrane shedding decreases the number of surface receptors during maturation, in addition to reduced synthesis and internalization, thus contributing to the developmental modulation of the effects of Epo and other cytokines.

Apoptosis is known to involve PS externalization (Leventis and Grinstein, 2010). Using cultures of erythroid precursors, we have found that depletion of Epo during their maturation results in apoptosis that is preceded by increased externalization of PS (Freikman and Fibach, 2011). These results suggest that the PS externalization is involved in the apoptosis of erythroid precursors under physiological conditions and that increased PS externalization due to oxidative stress may be responsible to pathologically increased apoptosis leading to ineffective erythropoiesis such as in the myelodysplastic syndrome or thal. Thus, membrane shedding may confer on erythroid precursors two opposing effects concerning apoptosis, acceleration, and inhibition by removing of EpoR and exposed PS, respectively.

### Iron Uptake

Erythroid maturation involves Hb production that requires iron uptake and heme and globin synthesis. Iron uptake is mostly carried out by binding of the serum iron-carrying (holo-) transferrin with its surface receptor (TfR). These receptors too undergo a biphasic modulation during maturation: they peak on erythroblasts (Moura et al., 2015) and decrease afterward to disappear altogether from the mature RBCs. This TfR modulation correlates with the extent of iron uptake and Hb production. It should be mentioned, however, that other mechanisms, such as non–TfR-route, receptor-mediated, uptake of ferritin (Leimberg et al., 2003) and iron (Prus and Fibach, 2011), may complement the main, TfR, route. Membrane shedding may play a role in the disappearance of TfR (Pan et al., 1985), as well as the alternative pathways of iron uptake.

### Erythroblastic Islands

Macrophages are essential for erythropoiesis (de Back et al., 2014; Moura et al., 2015). During their early development, erythroid precursors surround a central macrophage, forming the erythroblastic island (Bessis and Breton-Gorius, 1962; Chasis and Mohandas, 2008). Developmental significance of this structure is not entirely clear; one possibility is that it facilitates iron uptake by direct contact and non-TfR route, at early stages of erythroid precursors. Indeed, we have shown that cocultures of erythroid progenitors and macrophages develop Hb-containing precursors in the absence of holo-transferrin (Leimberg et al., 2008). This is probably a transient phenomenon, characterizing specific stages in the erythroid precursor maturation, prior to the peak of TfR exposure, after which this interaction is dissociated. It has been suggested that surface adhesion proteins on erythroid precursors might link them to macrophages and their extracellular matrix (Chasis and Mohandas, 2008; Soni et al., 2008). This could be hypothesized that the external PS might have a similar effect by binding to macrophages carrying PS receptors. Membrane shedding, in addition to reduced synthesis and internalization, may reduce the exposure of the adhesion proteins and PS moieties and thus lessens the binding and facilitates the release of erythroid cells from the island as they mature. This possibility awaits experimental confirmation.

### Size Reduction

This process characterizes erythroid maturation in the bone marrow, as well as RBC senescence in the circulation (Jandl, 1996). It serves a functional adaptation generating mature RBCs that are small enough to pass through narrow capillaries. Size reduction also leads to a high surface-to-volume ratio (Renoux et al., 2019), promoting gas exchange between the RBCs and tissue cells. Membrane shedding might be either the cause or the outcome of size reduction. We reported that inhibition of PS externalization and shedding prevented size reduction in differentiating erythroid cells (Freikman and Fibach, 2011), favoring the first possibility.

### Organelle Expulsion

At the end of their terminal maturation, erythroid precursors lose all their organelles—the nucleus (in most mammals), mitochondria, ribosomes, Golgi apparatus, and endoplasmic reticulum. Some of these processes involve membrane shedding. Nuclear expulsion (enucleation) requires preliminary changes taking place during differentiation, such as cell cycle arrest, chromatin condensation, and nuclear polarization (pyknosis). Enucleation is preceded by rearrangement of the cytoskeleton and clathrin-dependent generation of vacuoles at the nuclear–cytoplasmic border. This process, which occurs in orthochromatic erythroblasts, produces two uneven cells: a reticulocyte and a pyrenocyte. The latter, which contains the expelled nucleus surrounded by a thin layer of cytoplasm and the plasma membrane, is rapidly engulfed by the macrophages of the erythroblastic islands. The exposed PS serves as an “eat me” signal for their elimination (Yoshida et al., 2005). Enucleated reticulocyte continues to mature, losing approximately 20% of its surface (Willekens et al., 2008) and any remaining membrane-bound cytosolic organelle by an autophagy/exosome-combined pathway (Blanc et al., 2005). This subject was reviewed in (Moras et al., 2017).

Another mechanism of removing cellular components is the clathrin-dependent invagination of the plasma membrane to form early endosomes that eventually fuse with the plasma membrane and are released as exosomes. This process was first observed in reticulocytes with respect to TfR shedding (Harding et al., 1983; Davis et al., 1986).

## The Roles of Membrane Shedding in the Circulation

### RBC Senescence and Clearance

During their circulation, RBCs are exposed to stress conditions: physical, upon squeezing through small capillaries; hyperosmotic, upon passing through the kidney medulla; and oxidative, in the lungs. These stress conditions affect the RBC composition and characteristics, leading to senescence and eventually clearance by phagocytosis by macrophages in the reticuloendothelial system (Bratosin et al., 1998). Normally, the average RBC life span in the circulation is 120 days, but under pathological conditions, the life is shortened considerably, causing hemolytic anemia. Senescence is associated with exposure of various membrane signals (senescence signals), which includes externalization of PS and reduced sialic acid and CD47, and binding of immunoglobulins and opsonins [for detailed review, see Bosman et al. (2008)]. These changes are recognized by macrophages as “eat me” signals; however, the relative importance of each signal to clearance under normal and pathological conditions is not known.

Membrane shedding might affect RBC senescence and clearance by opposing mechanisms: (I) delaying clearance by removing intracellular oxidizing compounds such as oxidized Hb and aggregated Band-3 and (II) enhancing clearance by removing CD47, a surface compound that prevents recognition by macrophages (Stewart et al., 2005).

## Summary

**FIGURE 1:**
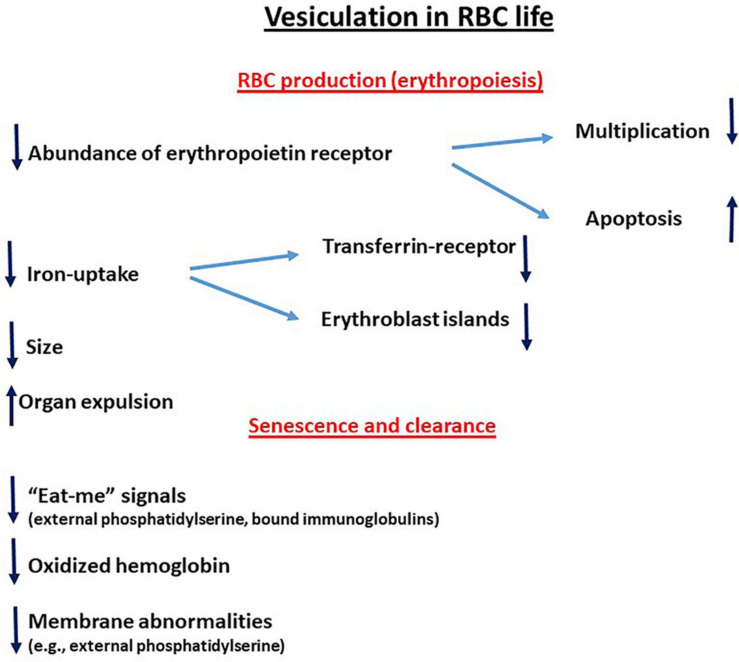
Vesiculation in RBC life. **↑** Upregulation; ↓downregulation.

**FIGURE 2:**
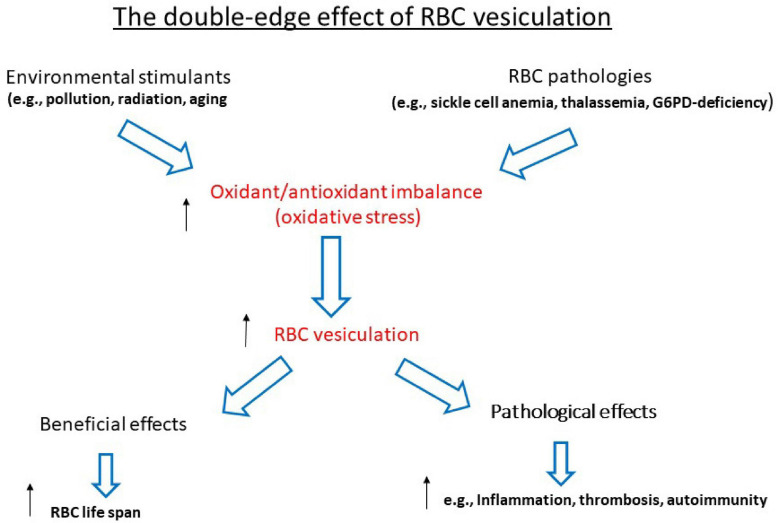
The double-edged effect of RBC vesiculation. ↑ Upregulation; ↓downregulation.

The reciprocal relationship between the redox state and membrane shedding is essential for the normal physiological functioning of cells and the abnormality during various pathologies. This review summarizes some aspects of this issue during the life of the RBC—their production, maturation, circulation, senescence, and, finally, clearance—to be replaced by new ones (graphically summarized in Figure 1). This relationship is ablated in the hemolytic anemias that involve the RBCs and their precursors as the major targets (graphically summarized in Figure 2). As the redox state and oxidative stress can be modulated by oxidants and antioxidants, treatment with such drugs might have potential benefits on membrane shedding. For example, L-glutamine supplementation by the oral powder Endari has recently been approved for use in SCA based on its antioxidant effects in RBCs (Kassa et al., 2019). The understanding of the multitude of effects of membrane shedding on different cellular functions and the therapeutic potentials of treatment of redox-modifying agents at present is highly hypothetical and awaits sound and detailed studies.

## Author Contributions

The author confirms being the sole contributor of this work and has approved it for publication.

## Conflict of Interest

The authors declare that the research was conducted in the absence of any commercial or financial relationships that could be construed as a potential conflict of interest.
